# Surgical Primary Tumor Resection Reduces Accumulation of CD11b^+^ Myeloid Cells in the Lungs Augmenting the Efficacy of an Intranasal Cancer Vaccination against Secondary Lung Metastasis

**DOI:** 10.3390/ph17010051

**Published:** 2023-12-28

**Authors:** Michael Donkor, Jamie Y. Choe, Danielle Marie Reid, Hope K. Fiadjoe, Byron Quinn, Amalendu Ranjan, Mark Pulse, Pankaj Chaudhary, Riyaz Basha, Harlan P. Jones

**Affiliations:** 1 Department of Microbiology, Immunology and Genetics, UNT Health Science Center, Fort Worth, TX 76107, USAdaniellereid@my.unthsc.edu (D.M.R.); hopefiadjoe@my.unthsc.edu (H.K.F.); amalendu.ranjan@unthsc.edu (A.R.); pankaj.chaudhary@unthsc.edu (P.C.);; 2 Department of Biology, Langston University, Langston, OK 73050, USA; 3 Department of Pharmaceutical Sciences, UNT Health Science Center, Fort Worth, TX 76107, USA; mark.pulse@unthsc.edu; 4 Institute for Health Disparities UNTHC 3500 Camp Bowie Boulevard, Fort Worth, TX 76107, USA

**Keywords:** lung metastasis, intranasal vaccination, immune response, nanoparticles, MDSCs

## Abstract

A hallmark of effective cancer treatment is the prevention of tumor reoccurrence and metastasis to distal organs, which are responsible for most cancer deaths. However, primary tumor resection is expected to be curative as most solid tumors have been shown both experimentally and clinically to accelerate metastasis to distal organs including the lungs. In this study, we evaluated the efficacy of our engineered nasal nano-vaccine (CpG-NP-Tag) in reducing accelerated lung metastasis resulting from primary tumor resection. Cytosine–phosphate–guanine oligonucleotide [CpG ODN]-conjugated nanoparticle [NP] encapsulating tumor antigen [Tag] (CpG-NP-Tag) was manufactured and tested in vivo using a syngeneic mouse mammary tumor model following intranasal delivery. We found that our nasal nano-vaccine (CpG-NP-Tag), compared to control NPs administered after primary mammary tumor resection, significantly reduced lung metastasis in female BALB/c mice subjected to surgery (surgery mice). An evaluation of vaccine efficacy in both surgery and non-surgery mice revealed that primary tumor resection reduces CD11b^+^ monocyte-derived suppressor-like cell accumulation in the lungs, allowing increased infiltration of vaccine-elicited T cells (IFN-γ CD8^+^ T cells) in the lungs of surgery mice compared to non-surgery mice. These findings suggest that the combination of the target delivery of a nasal vaccine in conjunction with the standard surgery of primary tumors is a plausible adjunctive treatment against the establishment of lung metastasis.

## 1. Introduction

The lung is a common site of most malignant tumor metastasis and accounts for the highest cancer mortality rates [1,2]. Such high mortalities can be attributed to the sewing of metastatic niches in the lung during the very early stages of tumor development even prior to primary tumor diagnosis [3,4,5,6]. The permissiveness of the lung and the other metastatic sites is in part due to immunosuppressive influences initiated by the primary tumor (e.g., breast tumor) that prepares the lung’s microenvironment for the evasion of malignant cells despite elevated inflammatory conditions initiated in response to tumor development [7,8,9,10,11]. A key process that facilitates the formation of this convivial metastatic environment to support lung metastasis is the propagation of CD11b^+^ myeloid-derived suppressor cells (MDSCs) into the lungs. These cells are notorious for promoting lung metastasis by secreting copious amounts of matrix metalloproteinase-9 (MMP-9), causing blood vessel remodeling that allows circulating tumor cell extravasation and promotion of inflammatory and immune suppressive microenvironments to support the growth of disseminated tumor cells [12,13]. Thus, the dynamic interaction between disseminated tumor cells and immune cells within the lung’s microenvironment is a critical determinant for the occurrence of overt lung metastasis.

Increasing evidence supports the metastatic-promoting influence of the surgical removal of primary tumors, which is intended to be curative for most solid tumors [14,15,16]. This has been attributed mainly to the increased shedding of tumor cells into circulation during surgery, an increased inflammatory response to surgical injury, and a significant suppression of antitumor immune responses post-surgery [17,18,19]. Targeting and reprogramming the lung microenvironment to increase tumor immunosurveillance presents an attractive opportunity to improve anticancer immunotherapy by halting secondary lung metastasis. Over the years, cancer vaccines have been used to induce antitumor immunity within the tumor microenvironment by stimulating endogenous T cells to attack tumor cells [20,21,22]. Accordingly, we have recently engineered an intranasal cancer vaccine (CpG-NP-Tag), utilizing targeted nano-vaccine delivery to pre-empt secondary lung metastasis by priming and boosting antitumor immunity in the lung [23]. We showed that our nasal nano-vaccine-induced localized mucosal-associated effector T cells and antibody immune responses were associated with a significant reduction in lung colonization by 4T1 cells, which are highly carcinogenic with high proclivity to metastasize to the lung in female BALB/c mice.

The object of this present study was two-fold. First, to test the efficacy of the nasal nano-vaccine as a preventative measure against lung metastasis using a spontaneous lung metastasis approach (e.g., mice with an existing primary mammary carcinoma). Secondly, we determined the sequelae of primary mammary tumor resection on lung metastasis in the absence of vaccination and the efficacy of our vaccine as a neoadjuvant to mitigating secondary lung metastases in the context of surgical resection. Our results demonstrate that primary mammary tumor resection increased lung metastatic growth of 4T1 tumors despite a reduction in CD11b^+^ MDSC-like cells in the lungs. In contrast, intranasal immunization of our nasal nano-vaccine significantly reduced metastasis in surgery mice. An analysis of the immune cell infiltration of the lungs of both non-surgery- and surgery-immunized mice revealed that nasal nano-vaccine immunization induced antitumor immunity in the lungs. Importantly, the decreased accumulation in CD11b^+^ MDSC-like cells after primary tumor resection was associated with the increased infiltration of IFN-γ-producing CD8^+^ T cells in the lungs in response to nasal nano-vaccine immunization. In sum, these findings support the efficacy of preemptive nasal vaccination as an approach to overcome exacerbated lung metastasis induced by the surgical resection of an existing primary tumor potentially orchestrated independent of the MDSC-like cell response.

## 2. Results

### 2.1. Primary Mammary Tumor Resection Accelerates Secondary Lung Metastasis

**Figure 1:**
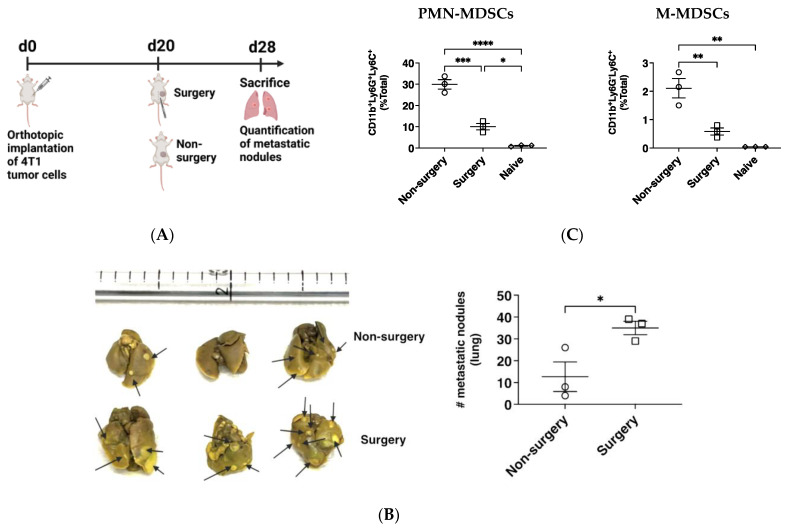
Primary mammary tumor resection increases secondary lung metastasis despite lower frequencies of CD11b^+^ myeloid suppressor-like cells in BALF: (**A**) Schematic of experimental timeline for spontaneous lung metastasis model in mice with intact primary tumor (non-surgery) or mice with primary tumor resection (surgery) mice. (**B**) Representative images of lungs from non-surgery or surgery mice. Quantification of metastatic lung nodules after placing lungs in 10% Bouin’s solution. (* *p* = 0.0396). (**C**) Frequencies of CD11b^+^ MDSC-like cells (Ly6G^+^Ly6C^+^ for PMN-MDSCs and Ly6G^−^Ly6C^+^ for M-MDSCs) among total cells isolated in BALF; (* *p* = 0.0150; ** *p* ≤ 0.005; *** *p* = 0.0002; **** *p* < 0.0001). *n* = 3; One-way ANOVA; Tukey’s multiple comparison test.

Initial studies were carried out to confirm the effect of surgery on lung tumor cell metastasis. Female BALB/c mice received orthotopic implantation of the murine mammary adenocarcinoma cell line (4T1). Subsequently, the solid tumor mass was surgically removed 20 days after implantation (Figure 1A). We confirmed the presence of lung metastasis in both mice with intact primary tumors (non-surgery mice) and mice that had undergone primary tumor resection (surgery mice) at 28 days PTI. Surgery mice had a significant (*p* ≤ 0.05) increase in lung metastatic growth, demonstrated by a greater number of lung metastatic nodules compared to mice with intact primary tumors (non-surgery) (Figure 1B). Accompanying an increase in lung metastasis among surgery mice was a significant (*p* ≤ 0.05) reduction in the percentage of CD11b^+^ polymorphonuclear MDSC-like cells (PMN-MDSCs; Ly6G^+^Ly6C^+^) and mononuclear MDSC-like cells (M-MDSCs; Ly6G^+^Ly6C^−^) detected in the bronchioalveolar lavage fluid of surgery mice compared to non-surgery mice (Figure 1C). 

### 2.2. CpG-NP-Tag Nasal Delivery Prevents Lung Metastasis among Mice Subjected to Surgical Resection of Primary Tumors

**Figure 2:**
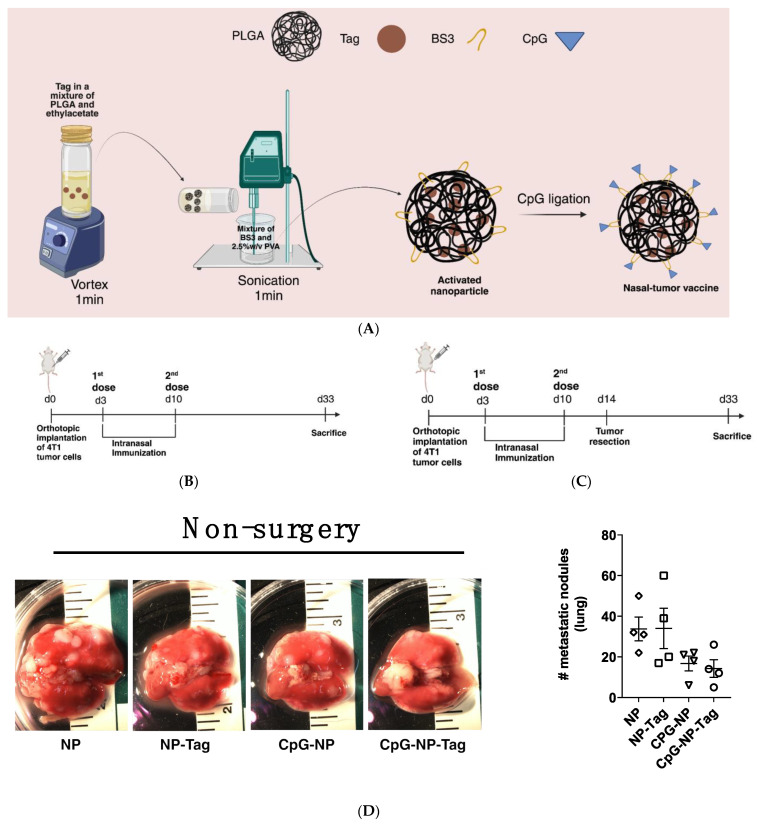

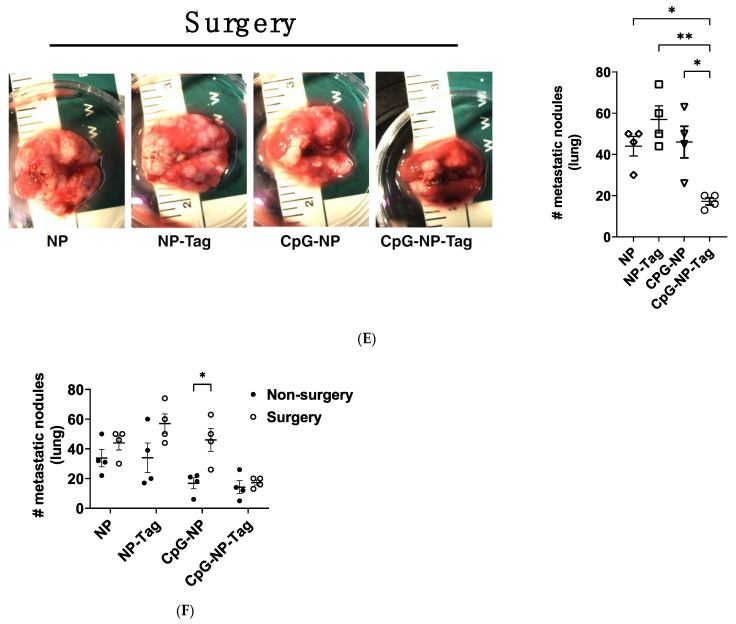
Efficacy of intranasal nano-vaccine immunization in preventing secondary lung metastasis in non-surgery and surgery mice: (**A**) Schematic of nasal tumor vaccine engineering. (**B**) Schematic of vaccine dosing in mice with intact primary mammary tumors (non-surgery mice). (**C**) Schematic of vaccine dosing in mice with primary tumor resection (surgery mice). Vaccine was administered prior to tumor resection after establishing mammary tumors in the mammary fat pad. (**D**,**E**) Representative images of lungs from non-surgery- (**C**) or surgery-immunized mice (**D**) and quantification of metastatic nodules in lungs. The number (#) of metastatic nodules is representative of counting performed on the whole lung and not only those represented by lung images (* *p* < 0.03; ** *p* = 0.0019). *n* = 4; (**D**,**E**) the mean number of metastatic nodules was analyzed using One-way ANOVA for comparison; Tukey’s multiple comparison test. (**F**) Quantification of metastatic nodules in the lungs of non-surgery and surgery mice. *n* = 4; Two-way ANOVA; Sidak’s multiple comparison test.

Having established that the surgical resection of an existing primary mammary tumor leads to increased lung metastasis, we evaluated the efficacy of our nasal nano-vaccine either alone or in combination with surgery to prevent secondary lung metastasis. Female BALB/c mice underwent the implantation of 4T1 tumors and were immunized intranasally with nasal nano-vaccine or respective NP controls (CpG-NP, NP-Tag, and NP) when tumors became palpable (day 3 PTI) and on day 10 PTI as a booster immunization (Figure 2B). Alternatively, primary tumor-bearing mice were subjected to surgical tumor resection of their primary tumor 4 days after booster immunization with either CpG-NP-Tag or NP controls (Figure 2C). At day 33 PTI, we observed no significant difference in lung metastatic growth across treatment groups in non-surgery mice (Figure 2D). However, a significant (*p* ≤ 0.05) decrease in lung metastatic nodules in CpG-NP-Tag-immunized surgery mice was observed in comparison with the respective controls, indicating an increased protection against lung metastatic growth (Figure 2E). By comparing the number of metastatic tumor nodules from the lungs of non-surgery mice to surgery mice, we observed an increased number of metastatic lesions in all mice subjected to surgery except mice immunized with CpG-NP-Tag (Figure 2F).

### 2.3. CpG-NP-Tag Nasal Immunization Promotes T Cell Infiltration in the Lungs following Surgical Resection of Primary Tumor

**Figure 3:**
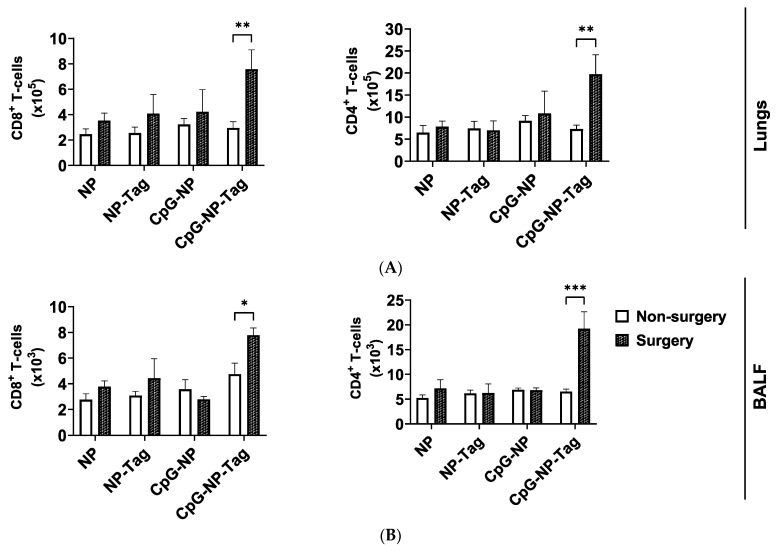
CpG-NP-Tag nasal administration increases both CD8 and CD4^+^ T cell frequencies in the lower respiratory of surgery mice: (**A**) The number of CD3^+^, CD8, and CD4 T cells that infiltrated the lungs of immunized non-surgery and surgery mice. (**B**) The number of CD3^+^, CD8, and CD4 T cells that infiltrated the BALF of immunized non-surgery and surgery mice; (* *p* < 0.05; ** *p* = 0.0096; *** *p* ≤ 0.0002). *n* = 4, Two-way ANOVA; Sidak’s multiple comparison test.

To understand why intranasal CpG-NP-Tag immunization can significantly reduce surgery-induced accelerated secondary lung metastasis, we assessed the effect of CpG-NP-Tag nasal immunization on the infiltration and effector functions of T cell in the lungs of NP-immunized mice for both non-surgery and surgery mice. Flow cytometry analysis confirmed that CpG-NP-Tag immunization significantly (*p* ≤ 0.05) increased the numbers of CD8^+^ and CD4^+^ T cells that infiltrated the lungs and BALF of surgery mice but not in non-surgery mice (Figure 3A,B). In contrast, the numbers of CD8^+^ and CD4^+^ T cells that infiltrated the lungs and BALF of control (CpG-NP, NP-Tag, and NP immunized) surgery mice and non-surgery mice were not significantly altered (Figure 3A,B).

### 2.4. CpG-NP-Tag Nasal Immunization Promotes Infiltration of IFN-γ-Producing CD8^+^ in the Lungs after Primary Tumor Resection

We next assessed T cell effector functions by stimulating lung-derived T cells from both non-surgery- and surgery-immunized mice with anti-CD3/CD28 antibodies. After stimulation, we recorded a significant increase in the proportions of CD8^+^ and CD4^+^ T cells that produced IFN-γ when mice immunized with CpG-NP-Tag were compared to mice immunized with respective NP controls (CpG-NP, NP-Tag, and NP) within both non-surgery and surgery conditions (Supplementary Figure S2B). In addition, we observed a higher proportion of GZMB-producing CD8^+^ T cells in CpG-NP-Tag-immunized mice but exclusively in non-surgery mice (Supplementary Figure S2C). However, the proportions of CD8^+^ and CD4^+^ T cells expressing CD69 remained unaltered by CpG-NP-Tag immunization in both non-surgery- and surgery-immunized mice (Supplementary Figure S2A). Together, we confirm that CpG-NP-Tag immunization led to an induction of T cell immune responses in both surgery and non-surgery-immunized mice based on higher proportions of CD8^+^ and CD4^+^ lung-infiltrating T cells producing IFN-γ after ex vivo restimulation.

**Figure 4:**
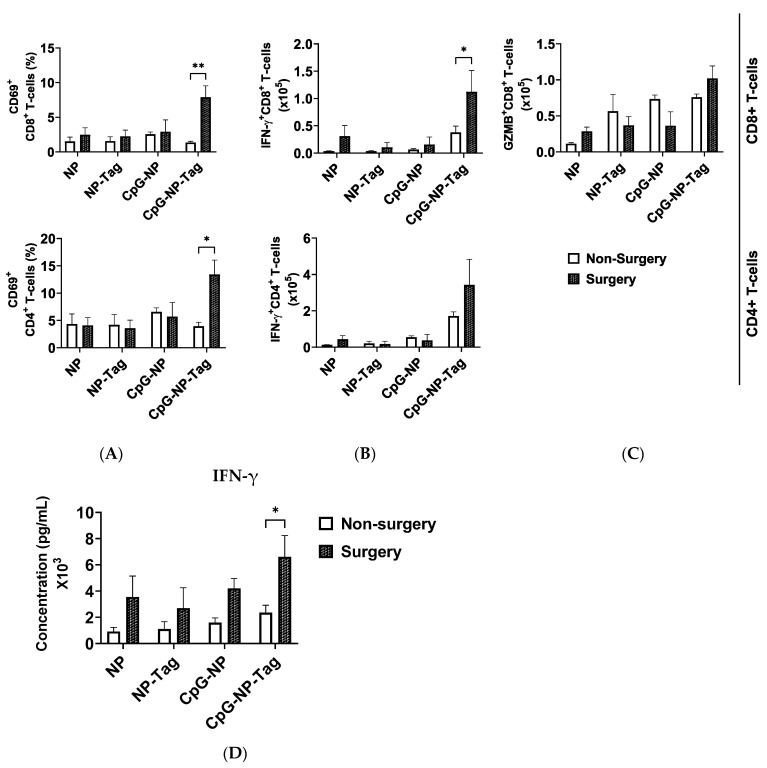
Nasal nano-vaccine increases IFN-γ-producing CD8^+^ T cell accumulation in the lungs of surgery mice: (**A**,**B**) The number of CD69-expressing (**A**) and IFN-γ-producing (**B**) cells among CD8^+^ and CD4^+^ T cells that infiltrated the lungs of both non-surgery- and surgery-immunized mice. (**C**) The number of granzyme B-producing CD8^+^ T cells that infiltrated the lungs of both non-surgery and surgery-immunized mice. (**D**) Concentration of IFN-γ in culture supernatants of non-surgery- and surgery-immunized mice after stimulation of lung leukocytes with anti-CD3/CD28 antibody using PBS as control. (* *p* < 0.05; ** *p* ≤ 0.0092). *n* = 4 Two-way ANOVA; Sidak’s multiple comparison test.

Having confirmed that CpG-NP-Tag intranasal immunization induced a T cell immune response in both non-surgery and surgery mice by comparing the proportions of activated and functional T cells in the lungs of the experimental mice, we next evaluated the effect of CpG-NP-Tag nasal vaccination on the infiltration of T cells with effector functions in the lungs of both non-surgery- and surgery-immunized mice. Specifically, we compared the numbers instead of the proportions of activated (CD69^+^) and functional (IFN-γ and GZMB production) CD8^+^ and CD4^+^ T cells that infiltrated the lungs of non-surgery and surgery mice following polyclonal restimulation with anti-CD3/CD28 antibodies. We observed a significant (*p* ≤ 0.05) increase in the number of CD8 and CD4 T cells expressing CD69 in the lungs of CpG-NP-Tag vaccinated surgery mice compared to non-surgery, while, at the same time, the number of CD8 and CD4 T cells expressing CD69 in the lungs of NP control vaccinated mice remained unaffected in non-surgery and surgery mice (Figure 4A). The number of IFN-γ-producing T cells was significantly (*p* ≤ 0.05) increased among CD8^+^ T cells but not CD4^+^ T cells in CpG-NP-Tag-vaccinated surgery mice compared to non-surgery vaccinated mice. However, the numbers of CD8 and CD4 T cells that produced IFN-γ remained unaltered across non-surgery and surgery mice immunized with NP controls (Figure 4B). Differences in GZMB-producing CD8+ T cells were not found in both CpG-NP-Tag and NP control, vaccinated mice (Figure 4C). The increase in the number of IFN-γ-producing CD8^+^ T cells that infiltrated the lungs of surgery CpG-NP-Tag-vaccinated mice was accompanied by a significant (* *p* ≤ 0.05) rise in the concentration of IFN-γ quantified in culture supernatants when lung leukocytes were stimulated for 4 days in the presence of anti-CD3/CD28 antibodies. (Figure 4D). Thus, nasal nano-vaccination promoted antitumor IFN-γ-producing CD8^+^ T cells in lungs counteracting the immunosuppressive effect of the surgical resection of the primary tumor.

### 2.5. Decreased Accumulation of CD11b^+^ MDSC-Like Cells in the Lungs Due to Surgery Increases Efficacy of Nasal CpG-NP-Tag

**Figure 5:**
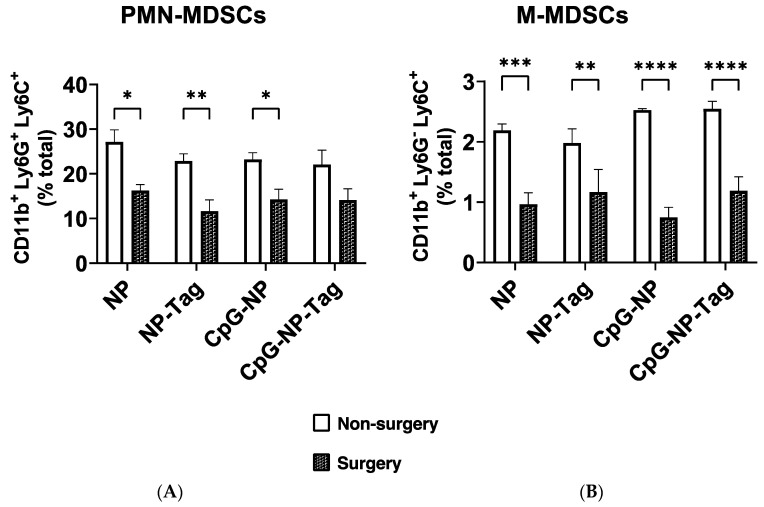
Primary mammary tumor resection led to a reduction in CD11b^+^ putative MDSCs in the lungs of immunized surgery mice: (**A**) Frequency of CD11b^+^ Ly6G^+^Ly6C^+^ (PMN-MDSCs) that infiltrated the lungs of non-surgery and surgery mice across immunized groups. (**B**) Frequency of CD11b^+^ Ly6G^+^Ly6C^−^ (M-MDSCs) that infiltrated the lungs of non-surgery- and surgery-immunized mice; (* *p* < 0.05; ** *p* < 0.005; *** *p* = 0.0002; **** *p* < 0.0001). *n* = 4; Two-way ANOVA; Sidak’s multiple comparison test.

Breast tumors establish metastasis at distal organs such as the lungs by propagating CD11b^+^ MDSCs to metastatic sites to create a convivial microenvironment to support lung metastasis by inducing inflammation and immunosuppression, as mentioned earlier. Several mechanisms via CD11b^+^ MDSCs are used to inhibit antitumor T cell responses including reducing T cell proliferation and inducing the apoptosis of T cells [24,25,26]. Given the significant (*p* < 0.05) reduction in the percentages of lung metastasis promoting CD11b^+^ MDSC-like cells in the bronchioalveolar lavage fluid of surgery-treated unimmunized mice compared to non-surgery unimmunized mice (Figure 1C), we hypothesized that vaccine efficacy is increased in surgery mice through the subversion of the primary tumor-induced infiltration of CD11b^+^ MDSC-like cells propagated to the lungs and allowing for the infiltration of antitumor IFN-γ-producing CD8^+^ T cells. We observed a significant (*p* ≤ 0.05) decrease in the accumulation of both PMN-MDSCs and M-MDSCs in the lungs of nasal nano-vaccinated surgery mice compared to non-surgery mice (Figure 5A,B).

## 3. Discussion

Most solid tumor deaths are a consequence of metastatic disease [27,28]. Therefore, primary cancer interventions aim to prevent the rise of metastasis by controlling primary tumor growth and preventing the seeding of tumor cells either through circulation or lymphatic spread to metastatic sites. There is increasing evidence that tumor cell dissemination to distal organs from primary tumors occurs early during tumor development; thus, research efforts are now focused on strategies to enforce the rejection of disseminated tumor cells (DTCs) at metastatic sites [5,29]. This has led to significant progress towards understanding the mechanisms governing the survival and outgrowth of DTCs. Previous studies have shown that primary tumors impact and remold the microenvironment of secondary organs by nurturing the formation of corroborative metastatic niches characterized by immunosuppression that allow DTCs to evade the hostile antitumor immune defenses leading to their outgrowth. Thus, developing strategies to restore antitumor machinery at metastatic sites to enforce the rejection of DTCs has become an urgent clinical need. One plausible strategy yet to be fully tested is direct inoculation at metastatic sites. Such an approach holds promise to improve vaccine effectiveness by pre-empting the generation of the immunosuppressive local microenvironment created by the existing primary tumor. At diagnosis of most extra-pulmonary primary tumors, dissemination to the lung would have occurred [5,30], increasing the chance of developing lung metastasis and significantly reducing survivorship in cancer patients. Capitalizing on the well-established strategies to deliver drugs to the lungs; for example, through intranasal administration, we engineered a nasal nano-vaccine: CpG-coated nanoparticles encapsulating tumor antigens (whole cell lysate) from mammary tumor cells. We previously showed that prior immunization (via intranasal instillation of nano-vaccine) prevented lung colonization through mammary tumor cells after intravenous injection [23]. In support, one study has shown that prior intranasal immunization with a melanoma cancer vaccine prevented lung colonization through melanoma cells after a challenge using a tail vein injection [31]. However, no study has evaluated the efficacy of intranasal cancer vaccine in preventing lung metastasis in the presence of an existing primary tumor with the biological progression from primary tumor to metastatic lung lesion and in the context of surgery-induced metastasis. Testing the efficacy of such an approach considers the immunosuppressive apparatus deployed to the lung by the primary tumor to ensure the survival of DTCs.

In this present study, we ascertained the translational efficacy of our nasal nano-vaccine in preventing lung metastasis by mimicking a clinical course of action. Specifically, we tested the potential of our nasal nano-vaccine at hindering lung metastasis after primary tumor resection, having established in this study that primary mammary tumor resection aggravates lung metastasis in the absence of any intervention. Our findings show that the nasal nano-vaccine could be part of an integrated cancer therapy used in combination with the standard surgical removal of the primary breast tumors to stem lung metastasis.

T cells are hallmarks for mediating antitumor immune responses in opposition to tumor cells that disseminate into the lungs [7,9,32]. In support, we have previously shown that CpG-NP-Tag nasal vaccination increases the frequency and effector functions of T cells specific for Tag in the lungs [23]. Therefore, to evaluate the efficacy of CpG-NP-Tag nasal vaccination in both non-surgery- and surgery-immunized mice, we assessed the effect of CpG-NP-Tag nasal vaccination on T cell infiltration and effector functions in the lungs of vaccinated mice, including both non-surgery and surgery mice. Our findings show that CpG-NP-Tag nasal vaccination induced antitumor T cell-mediated immune responses in both non-surgery and surgery mice. However, the number of CD8^+^ T cells infiltrating the lungs of non-surgery mice was significantly reduced, leading to a significant reduction in the number of IFN-γ-producing CD8^+^ T cells that infiltrated the lungs of non-surgery mice. These observations confirm that unresected primary tumors orchestrate mechanisms to reduce vaccine efficacy in the lungs by reducing the accumulation in vaccine-elicited T cells, particularly IFN-γ-producing CD8^+^ T cells, in the lungs. Our results also showed a higher proportion of GZMB-producing CD8^+^ T cells in the lungs of non-surgery mice compared to surgery mice that were immunized with the nasal tumor vaccine. This could have been because of the negative effects of surgery on the immune system and the need to combine the nasal tumor vaccine with other immunotherapies such as checkpoint inhibitors or bispecific antibodies that are targeted at activating exhausted T cells.

The presence of a primary breast tumor is associated with a rise in the accumulation of CD11b^+^, MDSCs, and Tregs, which exploits other mechanisms that cause the exhaustion of T cells in the lungs [25,33,34,35]. Here, our results indicate a significant increase in Tregs in non-surgery mice that were immunized with CpG-NP-Tag (Supplementary Figure S6). Notably, PD-1 expression, which denotes an exhausted T cell phenotype, was not altered in both non-surgery and surgery mice (Supplementary Figure S6). This observation decreases the likelihood that both Tregs and T cell exhaustion are possible mechanisms for decreased vaccine efficacy in favor of CD11b^+^ MDSC-like cells, which were increased in all non-surgery-immunized mice compared to their surgery counterparts, denoting that nasal tumor vaccination did not affect the levels of CD11b^+^ MDSC-like cells. Our findings could support a new paradigm by which CD11b^+^ MDSCs facilitate the reduced infiltration and accumulation of vaccine-elicited T cells within the lungs. Yet, more experiments are needed to clearly define the phenotype of these cells.

In summary, our work expands on the critical role cancer vaccines can play in preventing tumor recurrence at metastatic sites following primary tumor resection and provides new considerations for improving the effectiveness of cancer vaccines. For example, the detrimental effects of surgery (on any existing antitumor immune response present at the time of surgery) may necessitate that another dose of the vaccine be given post-surgery and at specific regular intervals throughout the life of the patient to ensure the patient has a cancer-free life. Future studies will be required to define the mechanisms employed by CD11b^+^ MDSCs that prevented vaccine-induced T cells from accumulating in the lungs after nasal nano-vaccine immunization. This will provide further insights into understanding the indirect mechanisms tumors use to evade the immune response leading to approaches that increase cancer vaccine efficacy, especially in situations where surgical resection is not possible (for example, in liquid cancers).

**Figure 6:**
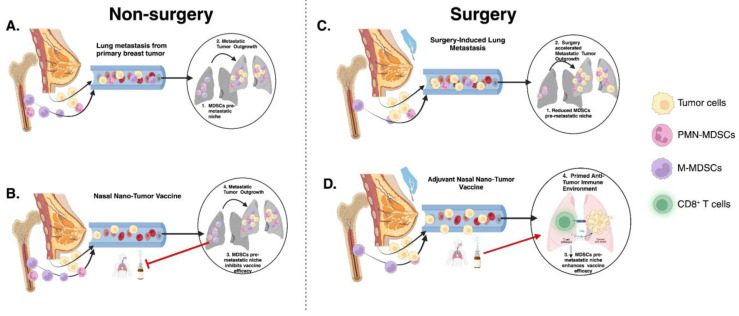
Schematic illustration of nasal nano-vaccine efficacy in non-surgery and surgery conditions: (**A**) Primary breast tumor induces propagation of MDSCs into the lungs to promote metastasis. (**B**) MDSCs pre-metastatic niche inhibits nasal nano-vaccine efficacy at preventing lung metastasis. (**C**) Primary breast tumor resection reduces pre-metastatic MDSCs but aggravates lung metastasis. (**D**) Nasal nano-vaccine inhibits surgery-enhanced lung metastasis due to a reduction in pre-metastatic MDSCs.

Collectively, our findings identify CD11b^+^ MDSCs as an essential determinant of intranasal vaccine efficacy in the lungs (Figure 6).

## 4. Materials and Methods

### 4.1. Materials and Reagents

Cell line (4T1) was sourced from the American Type Culture Collection (ATCC, Manassas, VA, USA). Reagents acquired from Sigma–Aldrich (St. Louis, MO, USA) include PLGA (Rosomer RG 505, ester terminated); inherent viscosity 0.61–0.74 dl/g; molecular weight (mw) 54,000–69,000 and Polyvinyl alcohol; mw 30,000–70,000—87–90% hydrolyzed. CpG-ODN 1826 murine TLR9 agonist was sourced from InvivoGen (San Diego, CA, USA). BS3 crosslinker, RPMI 1640 media, fetal bovine serum (FBS), and pen-strep were purchased from Thermo Fisher Scientific (Waltham, MA, USA).

### 4.2. Formulation and Characterization of CpG-NP-Tag and Control NP Constructs for Nasal Delivery

Tumor antigen (Tag) was prepared as previously described [23] and then encapsulated into PLGA polymer by employing our double emulsion method [23,36,37], producing NP-Tag (Figure 2A). The nanoparticle (NP) formulation (NP-Tag) was washed three (3) times with distilled water and finally redispersed in sucrose solution (5% *w*/*v*) followed by lyophilization on the Labconco freeze dryer system (Kansas, MO, USA) and stored at −20 °C until CpG conjugation. For the ideal conjugation of CpG, CpG ligands and redispersed NPs (1:1000 *w*/*w* ratio) were incubated for 0.75–1 h on an orbital shaker followed by two (2) PBS washes to remove excess ligands generating nasal CpG-NP-Tag. CpG surface-conjugated NP (CpG-NP), tumor antigen-encapsulating NP (Tag-NP), and NP only were formulated as NP controls. The particle size, zeta potential, polydispersity index (PDI), encapsulation efficiency, and tumor antigen protein loading were measured as reported in our earlier publication (ref) (Table 1).

### 4.3. Animal Model

BALB/c female adult mice (8–10 weeks) acquired from Charles Rivers Laboratories were used for all studies. Mice were harbored at the animal facility at the UNTHSC. All procedures were performed in accordance with the IACUC protocol # 2021-0039 at UNTHSC.

### 4.4. Tumor Challenge and Intranasal Immunization

A total of 2 × 10^5^ 4T1 tumor cells in a volume of 50 μL of PBS and Matrigel in a 1:1 ratio was implanted into the fourth mammary fat pad of female BALB/c mice on day 0 (Figure 1A and Figure 2B,C). Intranasal immunization began on day 3 post-tumor cell implantation when primary tumors became palpable with an average tumor volume of 13.10 mm^3^ ± 5.33. Mice were randomly placed into 4 different groups and were administered with 2 doses the of either CpG-NP-Tag or control NP constructs (CpG-NP, NP-Tag, and NP) via intranasal instillation under light anesthesia (Isoflurane) in 50 μL PBS. Each mouse received 25 mg of NP equivalent to 112.5 μg of Tag. The second intranasal vaccine dose was administered 7 days after the first dose. Body mass and tumor dimensions (digital caliper) were measured every 2 days and 5 days, respectively, after the first intranasal dose. Tumor volume was calculated using the standard formula: $V=0.5\times L\times W^{2}$, where L is the length and W is the width of the tumor measured using the caliper. Mice were sacrificed 33 days post orthotopic tumor implantation (PTI).

#### Surgical Removal of Tumors

To evaluate the implication of primary tumor resection on lung metastasis, tumors were surgically removed from tumor-bearing mice 20 days after tumor establishment in the mammary fat pad (Figure 1A). For the evaluation of vaccine efficacy, primary tumors were surgically removed 4 days after the second intranasal administration of NP formulations in tumor-bearing mice (Figure 2B). The primary tumors were completely removed as previously described [38,39]. Buprenorphine (0.005 mg/kg; sc) was administered promptly after surgery and again 12 h later for pain management.

### 4.5. Flow Cytometry

#### 4.5.1. Measurement of T Cell Infiltration in Lungs and BALF

Lung leukocytes and BALF cells isolated as previously described [23] were stained for 1hr at 4 °C with anti-mouse CD45 APC (clone; 30-F11, Tonbo Biosciences, San Diego CA, USA), anti-mouse CD3 FITC (clone; 17A2, Tonbo Biosciences, San Diego, CA, USA), anti-mouse CD4 BV605 (clone; GK 1.5, Biolegend, San Diego, CA, USA), anti-mouse CD8 PE (clone; 2.43, Tonbo Biosciences, San Diego, CA, USA) and anti-mouse PD-1 PE-Cy5 (clone; 29F.1A12, Biolegend, San Diego, CA, USA).

#### 4.5.2. Measurement of T Cell Responses

Lung leukocytes (1 × 10^6^) were stimulated with CD3/CD28 anti-mouse antibodies (2.5 μg/mL ea.) in the presence of 1X brefeldin A (Biolegend) for 8–12 h prior to surface staining with anti-mouse CD45 Pacific blue (clone; S18009F, Biolegend, San Diego, CA, USA), anti-mouse CD3 APC (clone; 145-2C11, Tonbo Biosciences, San Diego, CA, USA), anti-mouse CD8 FITC (clone; 53–6.7, Biolegend, San Diego, CA, USA), anti-mouse CD4 BV605 (clone; GK 1.5, Biolegend, San Diego, CA, USA), anti-mouse CD69 PE/Cy7 (clone; H1.2F3, Invitrogen, CA), and anti-mouse CD103 BV711 (clone; BV711, Biolegend, San Diego, CA, USA). After two washes, cells were fixed in 2% paraformaldehyde and treated with permeabilization buffer (0.5% tween 20 in FACS buffer). Cells were stained with anti-mouse GzmB PE (clone QA18A28; Biolegend, San Diego, CA, USA) and anti-mouse IFN-γ APC-Cy7 (clone; B-XMG1.2, Biolegend, San Diego, CA, USA).

#### 4.5.3. Measurement of Myeloid-Derived Suppressor Cell (MDSC) Accumulation

Lung leukocytes were stained for 1hr at 4 °C with anti-mouse CD45 APC (clone; 30-F11, Tonbo Biosciences, San Diego, CA, USA), anti-mouse CD11b BV650 (clone; B-M1/70, Biolegend, San Diego, CA, USA), anti-mouse Ly6C BV421 (clone; HK 1.4, Biolegend, San Diego, CA, USA), and anti-mouse Ly6G PE/Cy7 (clone; IA8, Biolegend, San Diego, CA, USA). All flow data were acquired on the Cytek Aurora 4-laser flow cytometer (Cytek, CA, USA) and analyzed using FlowJo V 10.8.1.

### 4.6. ELISA

Lung leukocytes (5 × 10^5^) were stimulated in 96-well flat-bottom plates with CD3/CD28 anti-mouse antibodies using PBS as a control and cultured at 37 °C with 5% CO_2_. Four days following stimulation, the culture supernatants were carefully removed after pelleting the cells and stored at −80 °C until the determination of IFN-γ concentration using the mouse IFN-γ ELISA Kit (Invitrogen, MA, USA) according to the manufacturer’s guidelines.

### 4.7. Statistical Analysis

Statistical analyses were conducted as depicted in figure legends on GraphPad Prism software, v.9.5.0.

## Figures and Tables

**Table 1:** Characterization of CpG-NP-Tag and control NP formulations for nasal delivery.

NP Construct	Particle Size (nm ± SD)	Zeta Potential (mV ± SD)	PDI	Encapsulation Efficiency (%)	Protein (µg)/ NP (mg)
CpG-NP-Tag	224.2 ± 7.01	−7.3 ± 0.57	0.183	14.7 ± 5.78	4.5
CpG-NP	209.0 ± 3.69	−1.1 ± 0.38	0.054	−	−
NP-Tag	225.3 ± 5.57	−9.0 ± 1.12	0.207	14.7 ± 5.78	4.5
NP	209.9 ± 2.73	−0.9 ± 0.20	0.045	−	−

The data illustrated in Table 1 represents the mean ± SD of readings from 3 different NP constructs.

## Data Availability

The data presented in this study are available in the Supplementary Material.

## Supplementary Materials

The following supporting information can be downloaded at: https://www.mdpi.com/article/10.3390/ph17010051/s1, Figure S1: Intranasal CpG-NP-Tag did not affect primary breast tumor growth. Figure S2: Intranasal CpG-NP-Tag increases the fractions of functional CD8^+^ and CD4^+^ T cells in the lungs of both CpG-NP-Tag-immunized non-surgery and surgery mice. Figure S3. The proportions of functional CD4^+^ and CD8^+^ T cells in the lungs of both immunized non-surgery and surgery mice. Figure S4: Intranasal CpG-NP-Tag immunization increases effector functions of lung resident memory T cells. Figure S5: The effect of intranasal nano-vaccine immunization on TH2 cytokines production using CD4^+^ T cells. Figure S6: The effect of nano-vaccine immunization on the number of CD4^+^ Tregs and the expression levels of PD-1 on CD8^+^ and CD4^+^ T cells in the lungs of immunized non-surgery and surgery mice. Figure S7: Representative FACS blots of CD11b^+^ MDSC-like cells (Ly6G^+^Ly6C^+^ for PMN-MDSCs and Ly6G^−^Ly6C^+^ for M-MDSCs). Figure S8: Gating strategy to identify IFN-γ- and GZMB-producing T cells in the lungs of immunized non-surgery and surgery mice that produce IFN-γ. Figure S9: Characterization of nanoparticles.
