# Supplementary material for: Surgical Primary Tumor Resection Reduces Accumulation of CD11b+ Myeloid Cells in the Lungs Augmenting the Efficacy of an Intranasal Cancer Vaccination against Secondary Lung Metastasis
Source: Pharmaceuticals (Basel). 2023 Dec 28;17(1):51. doi: 10.3390/ph17010051 (PMC10821475; doi:10.3390/ph17010051)
Supplement: Supplementary file 1 [file pharmaceuticals-17-00051-s001.zip › pharmaceuticals-2776940-supplementary.pdf]

pharmaceutics

MDPI

Supplementary Information:

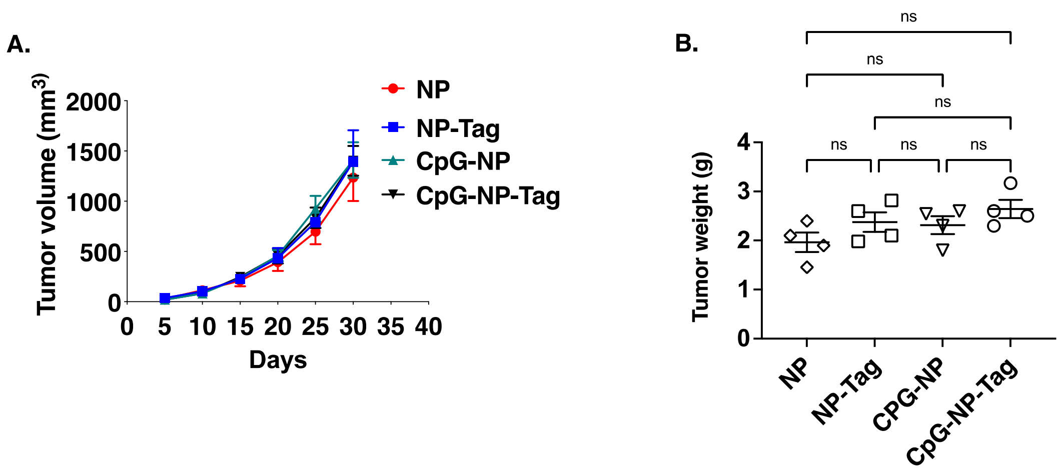

volume
ery mice
NOVA

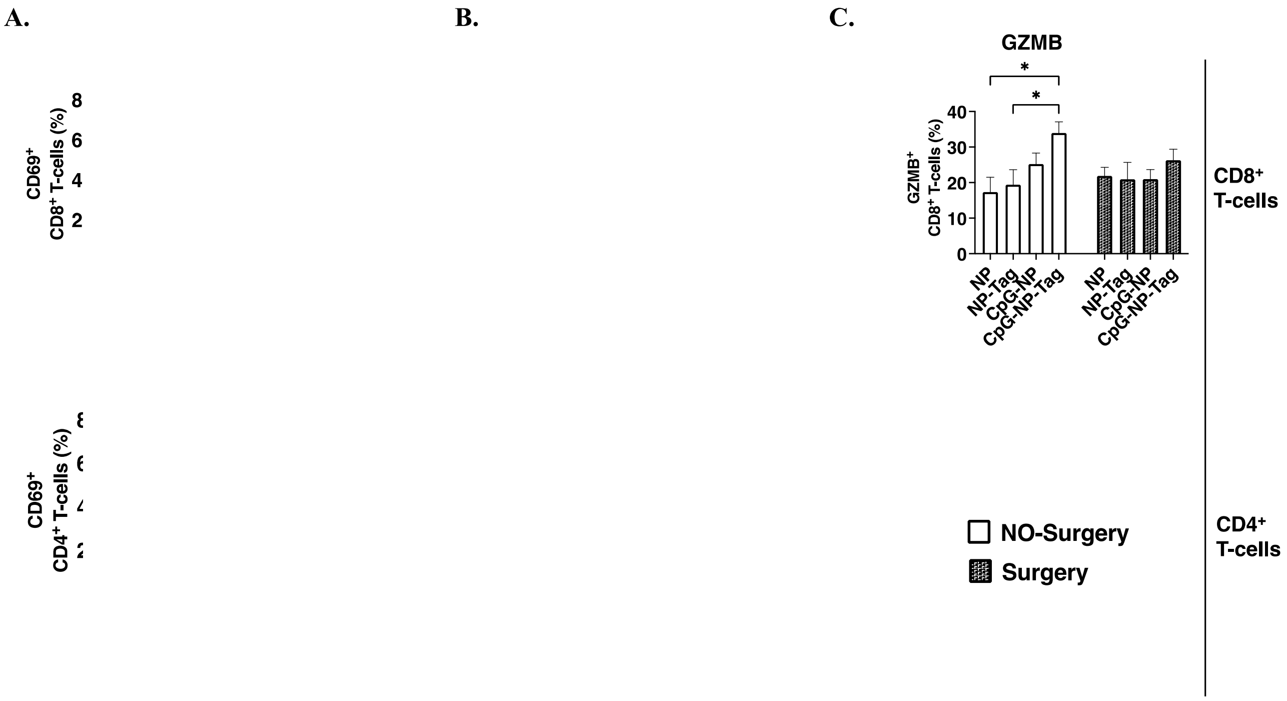

Supplementary Figure S2. Intranasal CpG-NP-Tag increases the fractions of functional CD8⁺ and CD4⁺ T cells in the lungs of both CpG-NP-Tag immunized non-surgery and surgery mice. (A,B) The fractions of CD69 expressing (A), and IFN-γ producing (B) cells among CD8⁺ (upper panels) and CD4⁺ (lower panels) T cells in the lungs of immunized non-surgery and surgery mice. **(C)** The fractions of GZMB producing cells among CD8⁺ T cells in the lungs of immunized non-surgery and surgery mice. (*p < 0.05; **p < 0.005; ***P < 0.0005). n = 4; Two-way ANOVA followed by Sidak's multiple comparison test.

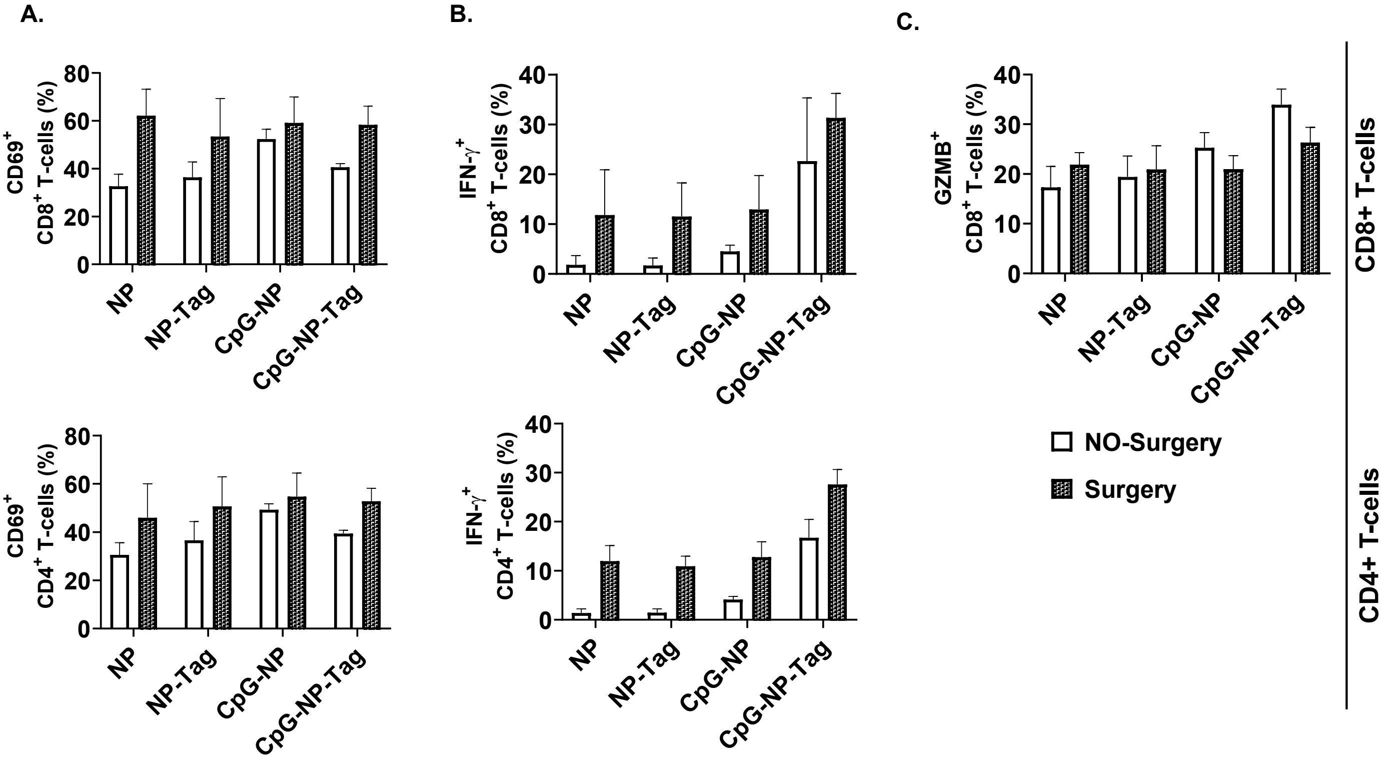

Supplementary Figure S3. The proportions of functional CD4+ and CD8+ T cells in the lungs of both immunized non-surgery and surgery mice. (A, B) The fractions of CD69 expressing (A) and IFN-γ producing (B) CD8+ (upper panels) and CD4+ (lower panels) T cells in the lungs of immunized non-surgery and surgery. **(C)** The fractions of GZMB producing cells among CD8+ T cells in the lungs of immunized non-surgery and surgery mice. n = 4; Two-way ANOVA; Sidak's multiple comparison test.

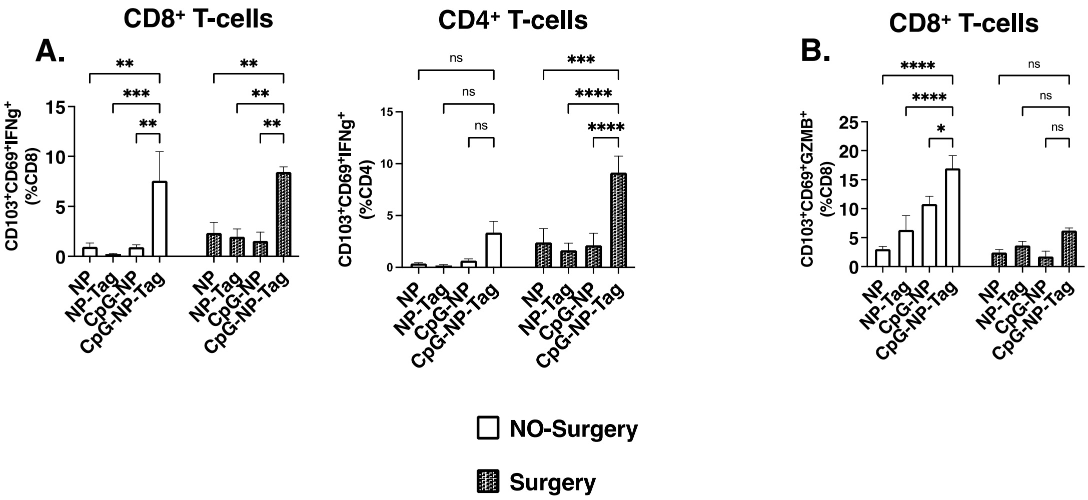

mory T cells.
ls. **(B)** Propor-
5; **p < 0.005;

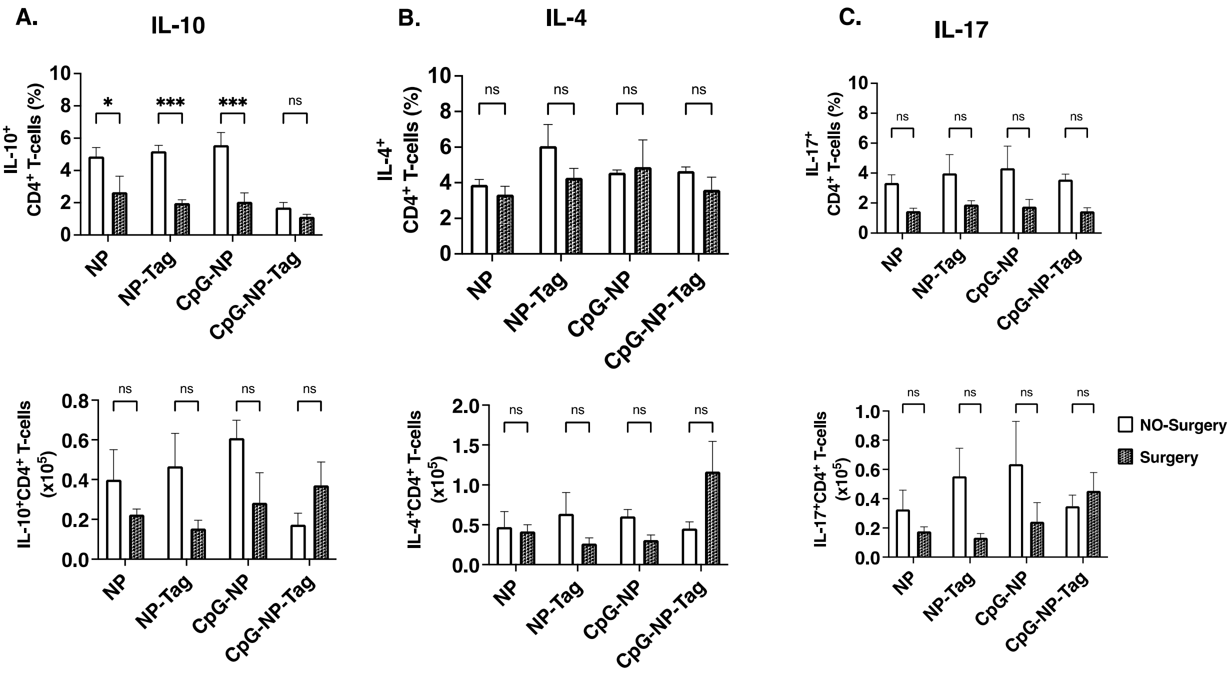

Supplementary Figure S5. The effect of intranasal nano-vaccine immunization on TH2 cytokines production by CD4+ T cells. (A,B,C) The fraction (upper panels) and quantity (lower panels) of IL-10 (A) , IL-4 (B) and IL-17 (C) producing cells among CD4+ T cells in the lungs of immunized non-surgery and surgery mice. (ns, not significant *p < 0.05; ***P < 0.0005;). n = 4; Two-way ANOVA followed by Sidak's multiple comparison test.

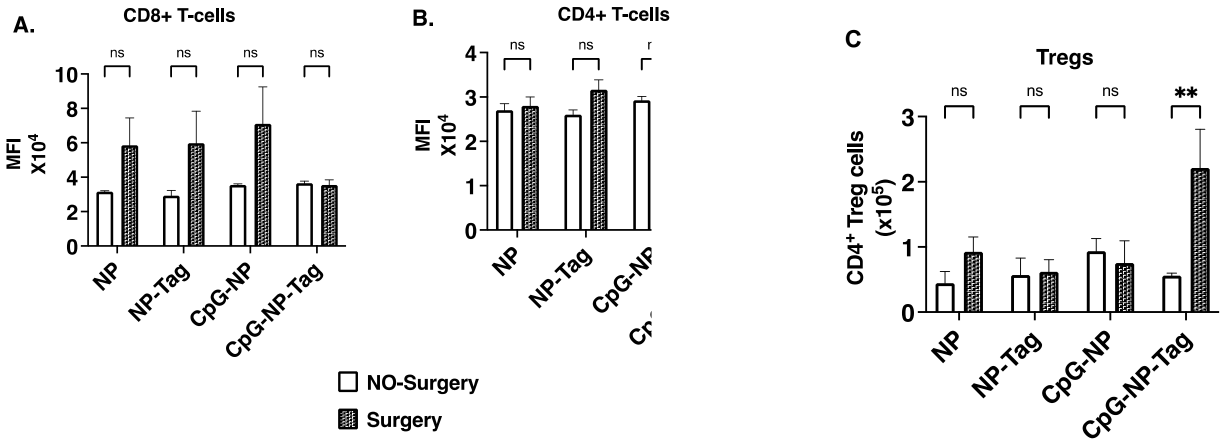

Supplementary Figure S6. The effect of nano-vaccine immu
PD-1 on $CD8^+$ and $CD4^+$ T cells in the lungs of immunized non-surgery and surgery mice. (A and B) The expression levels of PD-1 on $CD8^+$ (A) and $CD4^+$ (B) T cells. **(C)** The total number $CD4^+$ Tregs in the lungs of non-surgery and surgery immunized mice. (ns, not significant; $^{**}p < 0.005$) n = 4; Two-way ANOVA followed by Sidak's multiple comparison test.

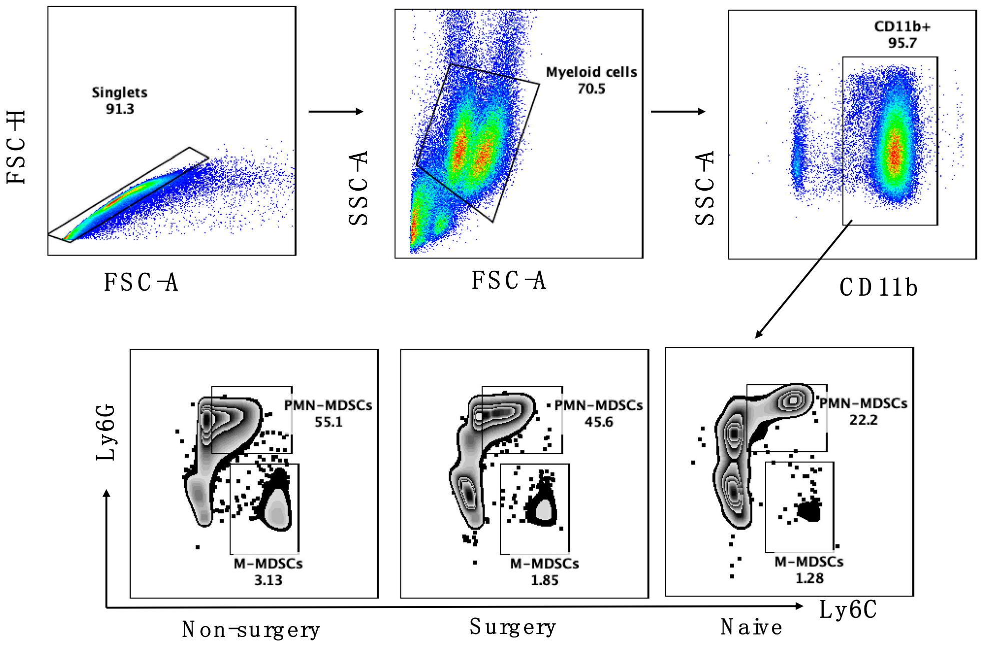

Supplementary Figure S7. Representative FACS blots of CD11b$^+$ MDSC-like cells (Ly6G$^+$Ly6C$^+$ for PMN-MDSCs and Ly6G$^-$Ly6C$^+$ for M-MDSCs).

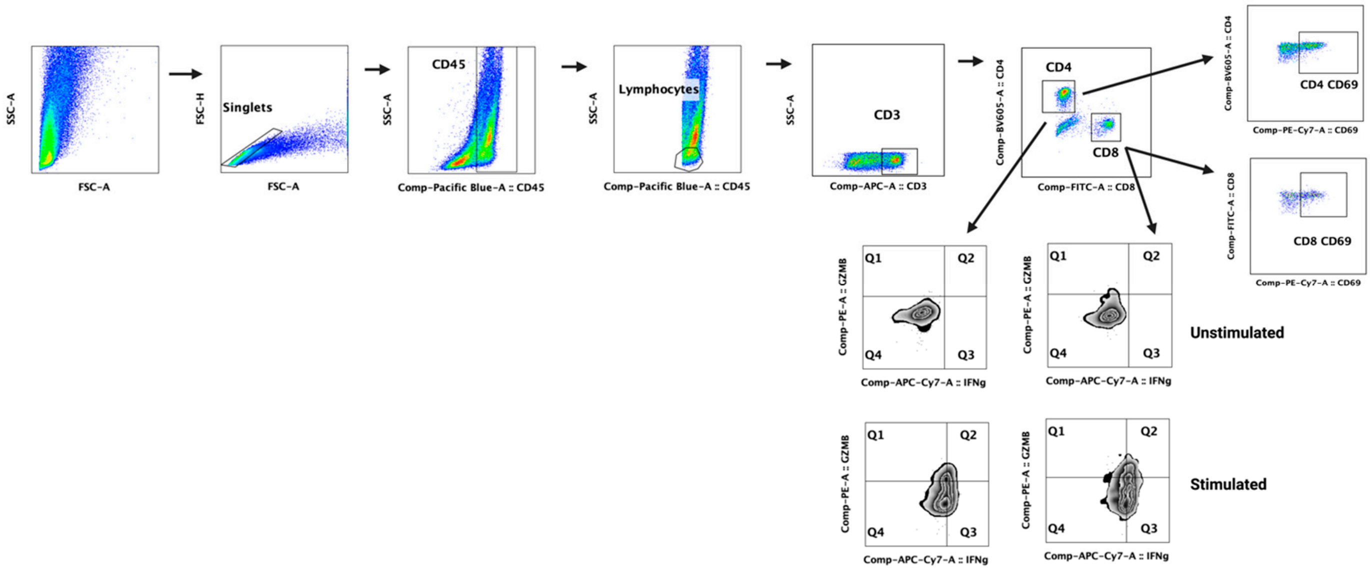

Supplementary Figure S8. Gating strategy to identify IFN-γ and GZMB producing T cells in the lungs of immunized non-surgery and surgery mice that produce IFN-γ.

A i

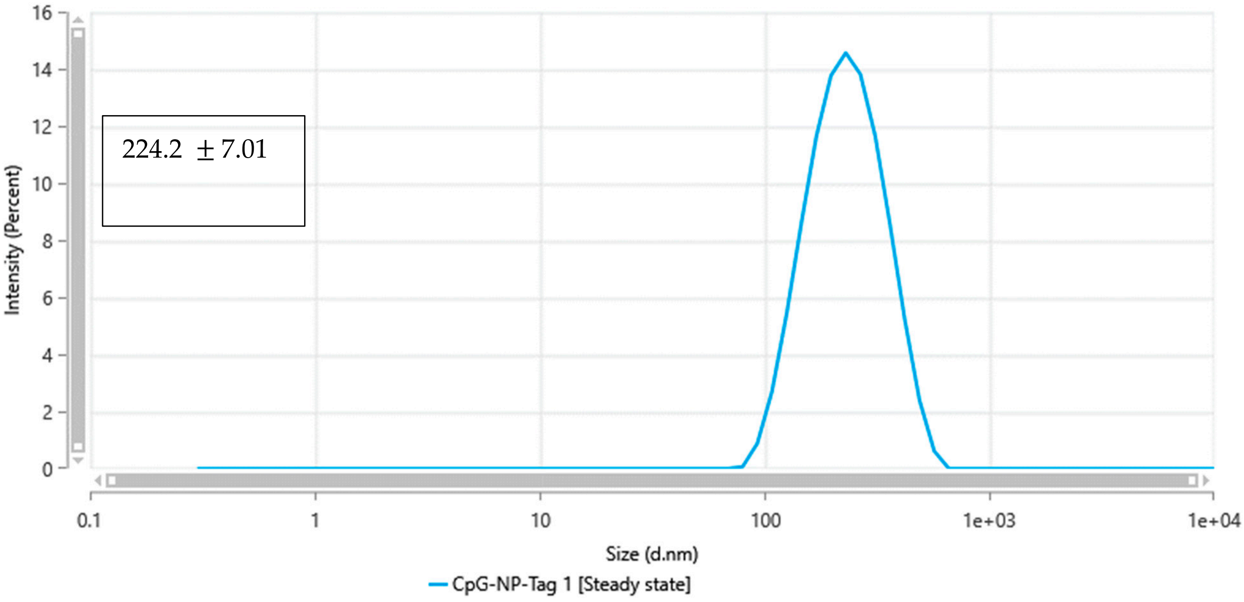
224.2 ± 7.01
Intensity (Percent)
16
14
12
10
8
6
4
2
0
0.1
1
10
100
1e+03
1e+04
Size (d.nm)
CpG-NP-Tag 1 [Steady state]

ii

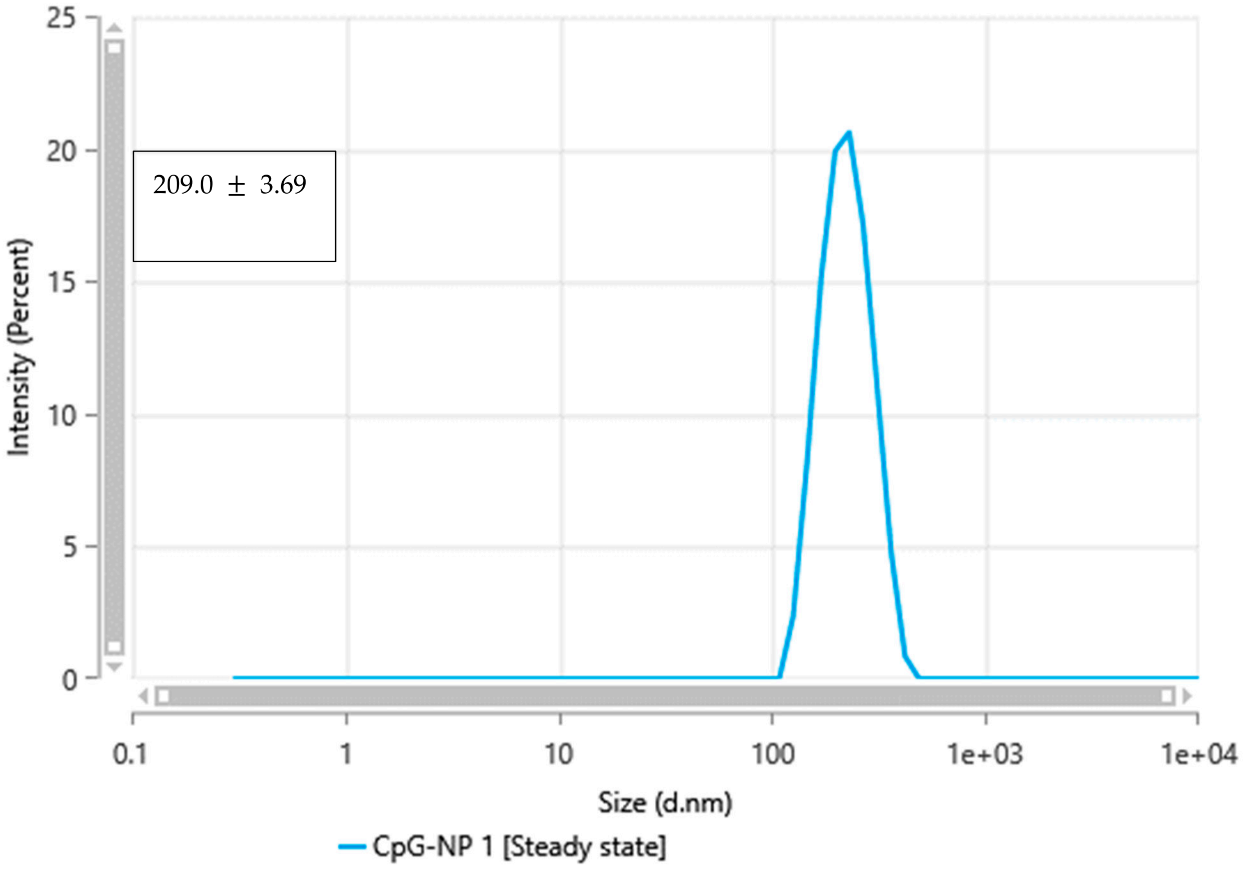
209.0 ± 3.69
Intensity (Percent)
25
20
15
10
5
0
0.1
1
10
100
1e+03
1e+04
Size (d.nm)
CpG-NP 1 [Steady state]

iii

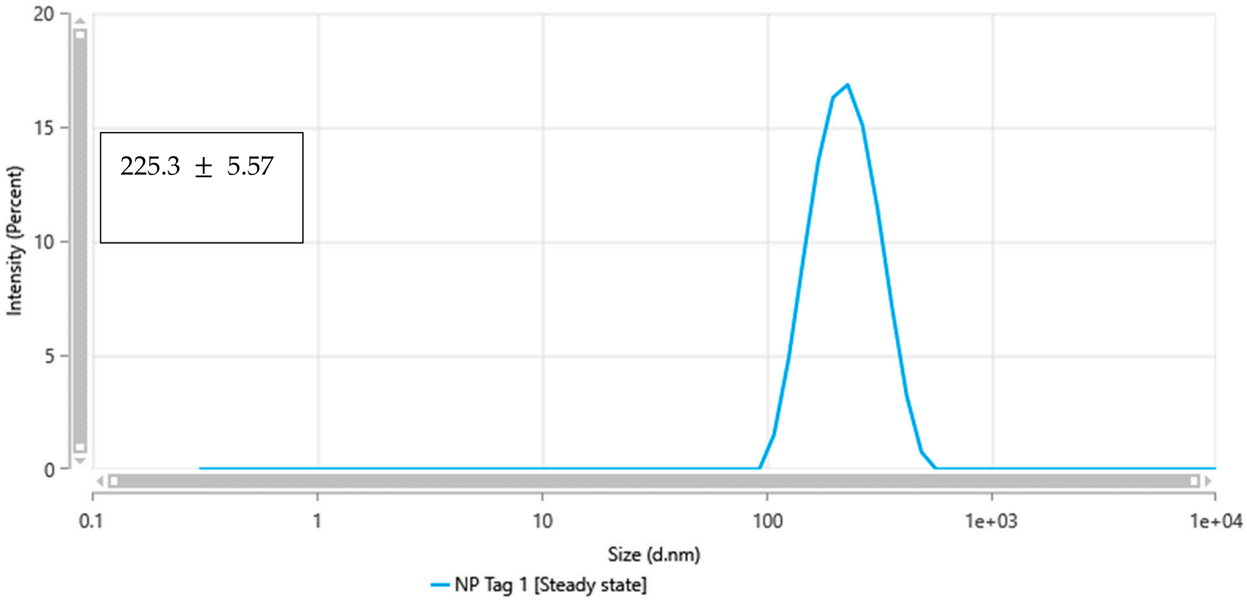
225.3 ± 5.57
Intensity (Percent)
20
15
10
5
0
0.1
1
10
100
1e+03
1e+04
Size (d.nm)
NP Tag 1 [Steady state]

iv

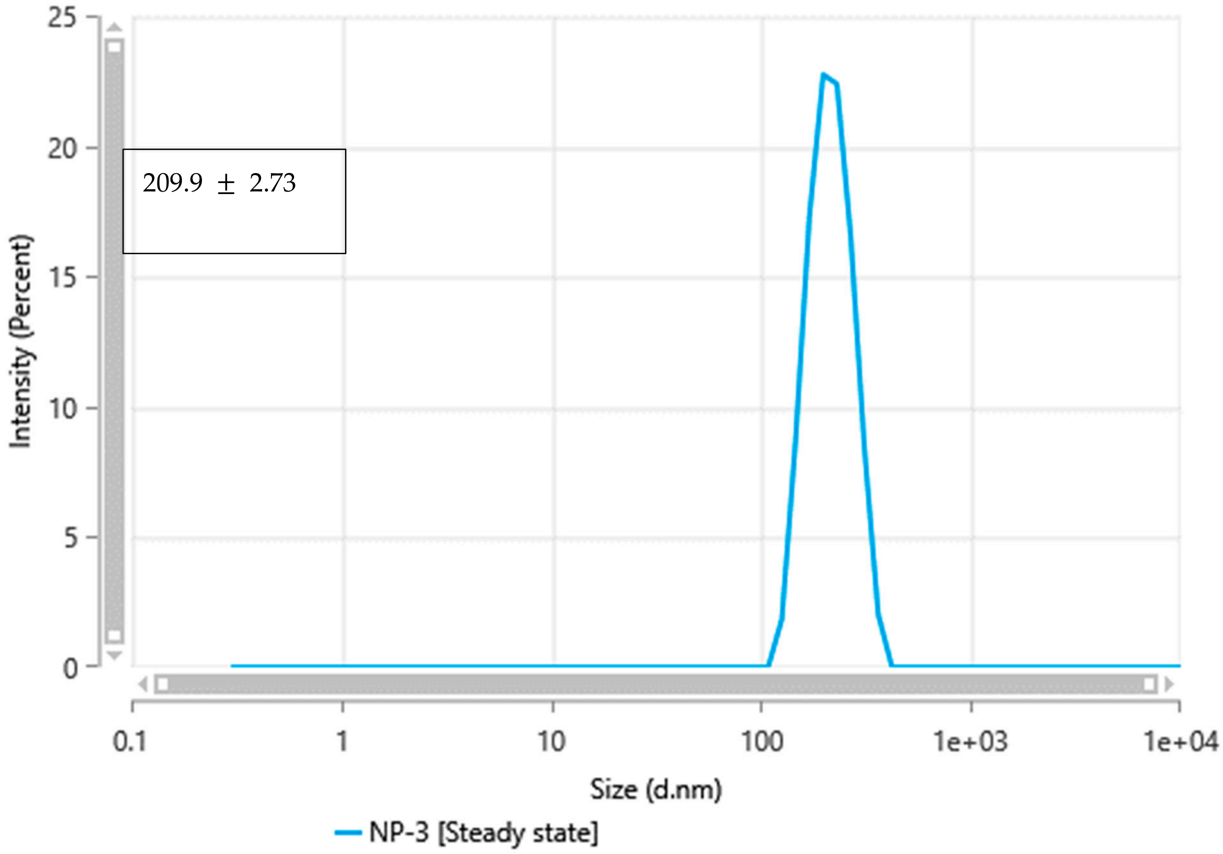
209.9 ± 2.73
Intensity (Percent)
25
20
15
10
5
0
0.1
1
10
100
1e+03
1e+04
Size (d.nm)
NP-3 [Steady state]

B i

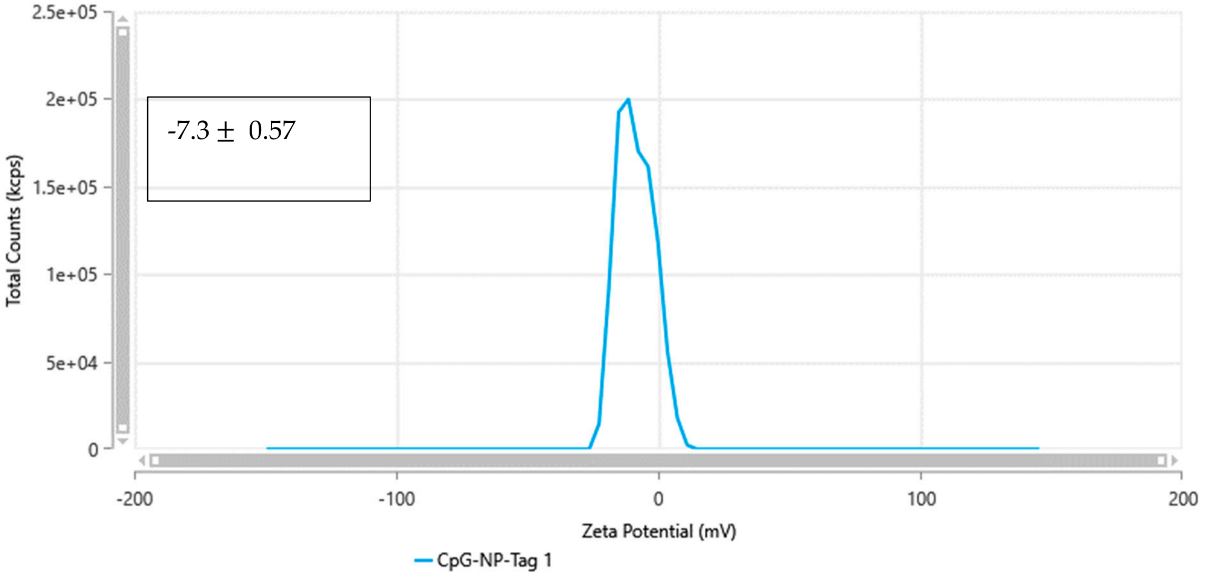
-7.3 ± 0.57
2.5e+05
2e+05
1.5e+05
1e+05
5e+04
0
Total Counts (kcps)
-200
-100
0
100
200
Zeta Potential (mV)
CpG-NP-Tag 1

ii

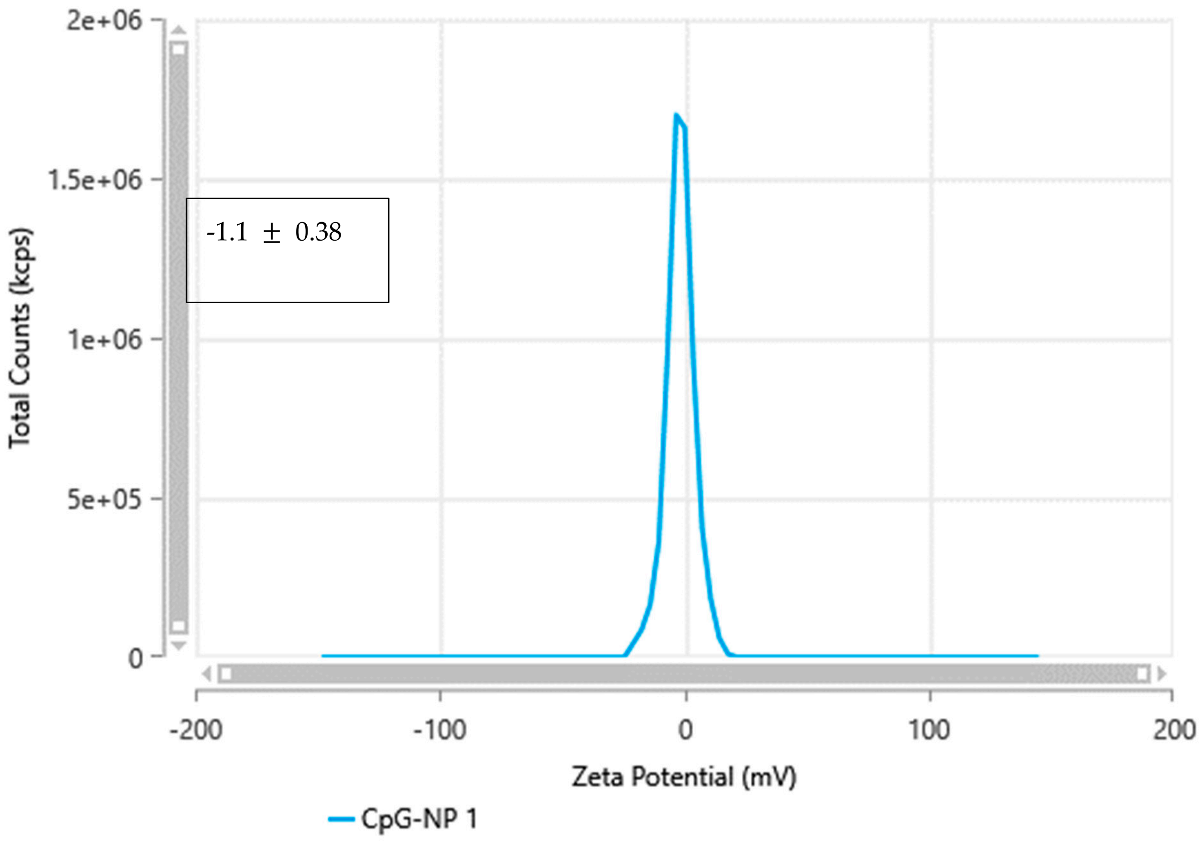
-1.1 ± 0.38
2e+06
1.5e+06
1e+06
5e+05
0
Total Counts (kcps)
-200
-100
0
100
200
Zeta Potential (mV)
CpG-NP 1

iii

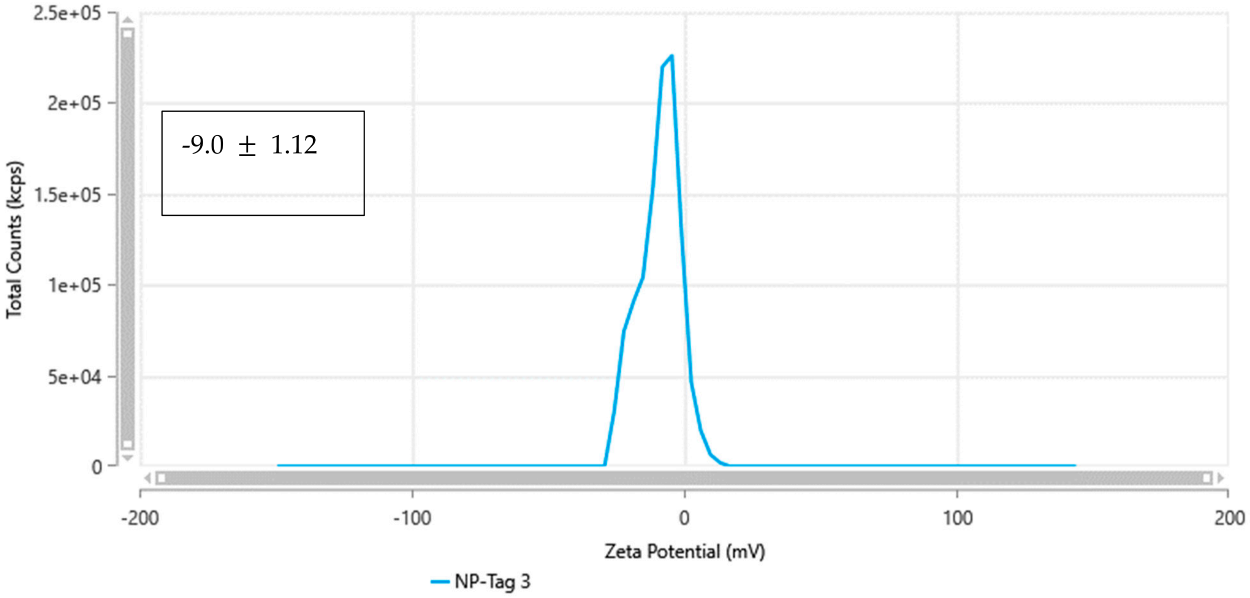
-9.0 ± 1.12
Total Counts (kcps)
2.5e+05
2e+05
1.5e+05
1e+05
5e+04
0
-200
-100
0
100
200
Zeta Potential (mV)
NP-Tag 3

iv

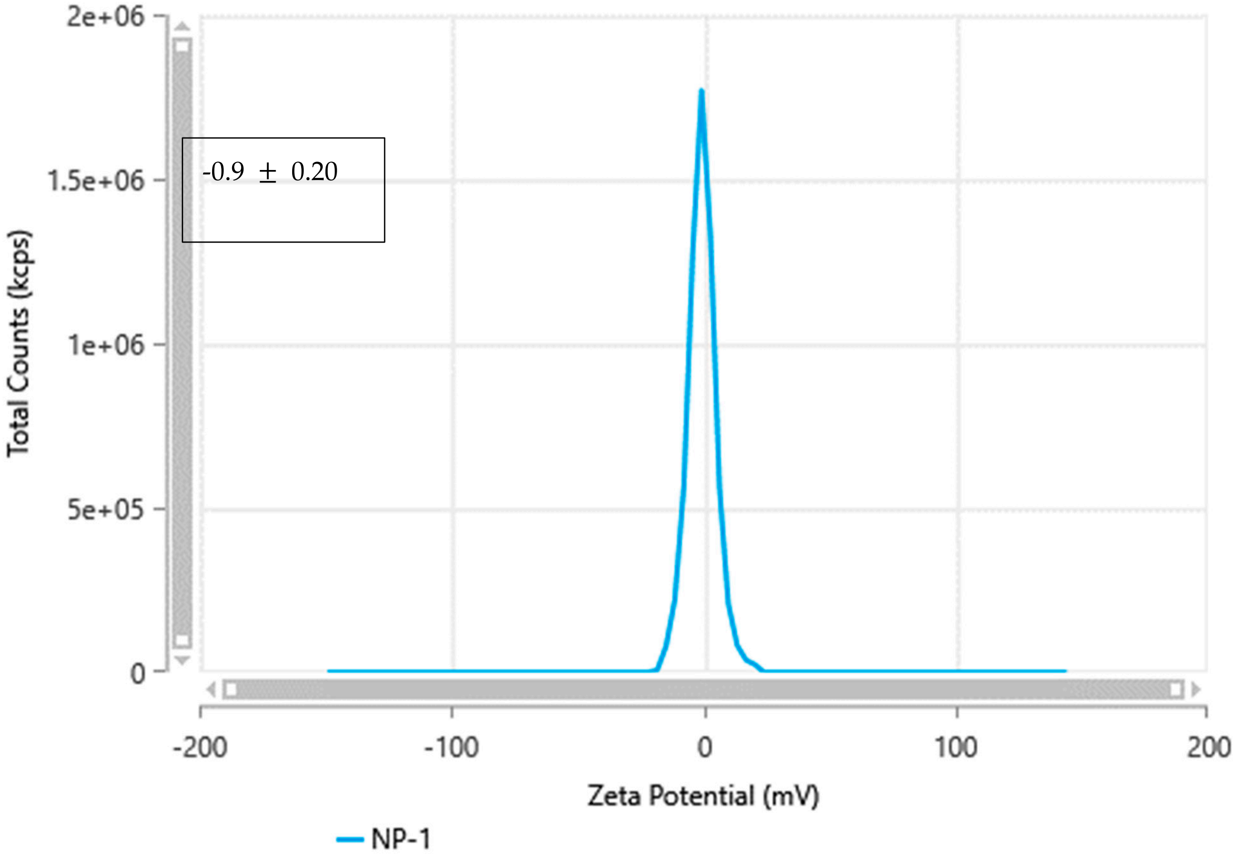
-0.9 ± 0.20
Total Counts (kcps)
2e+06
1.5e+06
1e+06
5e+05
0
-200
-100
0
100
200
Zeta Potential (mV)
NP-1

Supplementary Figure S9. Characterization of Nanoparticles. (A) Particle size distribution of CpG-NP-Tag **(i)**, CpG-NP **(ii)**, NP-Tag **(iii)** and NP **(iv)** derived from dynamic light scattering measurements. **(B)** CpG-NP-Tag **(i)**, CpG-NP **(ii)**, NP-Tag **(iii)** and NP **(iv)** derived from dynamic light scattering measurements.
